# Investigations into Potential Extrasynaptic Communication between the Dopaminergic and Nitrergic Systems

**DOI:** 10.3389/fphys.2012.00372

**Published:** 2012-09-25

**Authors:** M. Mitkovski, F. E. Padovan-Neto, R. Raisman-Vozari, L. Ginestet, C. A. da-Silva, E. A. Del-Bel

**Affiliations:** ^1^Light Microscopy Facility, Max-Planck-Institute of Experimental MedicineGöttingen, Germany; ^2^Department MEF-Physiology, Dental School of Ribeirão Preto, University of São PauloRibeirão Preto, Brazil; ^3^Neurology-Neurociences, Medical School, University of São PauloRibeirão Preto, Brazil; ^4^INSERM, UMR 975, CRICM, Thérapeutique Expérimentale de la NeurodégénérescenceParis, France; ^5^Faculté de Médecine, Université Pierre-et-Marie-CurieParis, France; ^6^CNRS, UMR 7225Paris, France

**Keywords:** dopamine, nitric oxide synthase, Parkinson disease, volume transmission, neurotransmitter spillover, synapse, plasticity, tyrosine hydroxylase

## Abstract

Nitric oxide is unconstrained by cell membranes and can therefore act along a broad distance as a *volume transmitter*. Spillover of nitric oxide between neurons may have a major impact on central nervous system diseases and particularly on neurodegeneration. There is evidence whereby communication between nitrergic and dopaminergic systems plays an essential role in the control of the nigrostriatal pathway. However, there is sparse information for either the coexistence or overlap of nitric oxide and dopaminergic structures. The dual localization of immunoreactivity for nitric oxide synthase (NOS) and tyrosine hydroxylase, enzymes responsible for the synthesis of nitric oxide and dopamine, respectively, was examined in neurons of the nigrostriatal pathway in the rat brain by means of a double-immunohistochemical method and confocal laser scanning microscopy, acquired at the resolution limit. After perfusional fixation, the brains were cut and double-immunostained. A proximity analysis of tyrosine hydroxylase and NOS structures was done using binary masks generated from the respective maximum projections, using confocal laser microscopy. Unrevealed regions were determined somatodendritic positive for both NOS and tyrosine hydroxylase, within an image limit resolution at 2 μm-wide margin. The described interconnected localization of nNOS(+) and TH(+) containing neuronal fibers and cells bodies in the nigrostriatal pathway propose a close anatomical link between the two neurotransmitters.

## Introduction

The mammalian basal ganglia (also referred to as caudate–putamen or neostriatum) are a group of subcortical nuclei implicated in a multiplicity of functions including motor, cognitive, and mnemonic behaviors (Bolam et al., 2000; Graybiel, 2000; Gerfen and Surmeier, 2011). The response of the basal ganglia circuitry to cortical stimuli is modulated by the neurotransmitter dopamine (Shen et al., 2008). Dopamine is crucial to motor, motivational, and reward-related functions of the central nervous system (Bolam et al., 2000; Graybiel, 2000). Degeneration of the dopamine system causes neurological disorders such as Parkinson’s disease and is involved in multisystem atrophy (Haavik and Toska, 1998; Nagatsu and Ichinose, 1999; Benavides-Piccione and DeFelipe, 2003).

**Figure 1:**
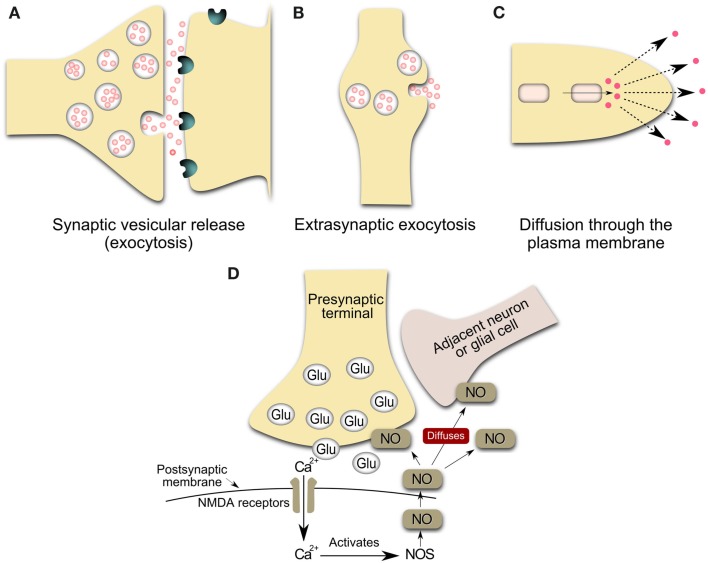
**Schematic representation of the main sources of volume transmission involving dopamine and nitric oxide signals in the central nervous system (Inspired by Zoli et al., 1999; Syková, 2004)**. **(A)** Synaptic transmission: Synaptic vesicular release followed by the diffusion of the transmitter outside the synaptic cleft at an effective concentration. Neurotransmitters can escape from the synaptic cleft (especially during repetitive stimulation and/or the massive release of a transmitter) and affect extrasynaptic receptors on glia or neurons or reach a neighboring synapse. These phenomena have been called “transmitter spillover” and “synaptic crosstalk.” This loss of synaptic independence means that transmitter release at one synapse can lead to the activation of receptors in nearby synapses. **(B)** Volume transmission: vesicular release from non-junctional varicosities, i.e. varicosities lacking pre-synaptic specializations and post-synaptic densities. Axons both in the central nervous system and in the autonomic nervous system form varicose (*boutons-en-passant*) branches that may release and uptake neurotransmitters. The transmitter release from varicosity without synaptic contact diffuses far away from the release site and activates remote receptors of high affinity. **(C,D)** Release of gaseous transmitters, e.g. nitric oxide release from neurons. The inside cell squares are representative of a local synthesis of the neurotransmitter, frequently by enzymatic regulated activity. Nitric oxide presumably operates within a 3-dimensional volume of tissue rather than, more conventionally, between immediately adjacent cellular partners (Kiss and Vizi, 2001). Glu, glutamate vesicles; NOS, nitric oxide synthase; NO, nitric oxide; NMDA, *N*-Methyl-d-Aspartate.

In addition to releasing dopamine from nerve terminals in striatum, nigrostriatal dopamine neurons synthesize and release dopamine from the soma and dendrites Geffen et al., 1976; Paden et al., 1976; Nieoullon et al., 1977; Wilson et al., 1977; Heeringa and Abercrombie, 1995). The molecular mechanisms underlying the modulatory role of dopamine on striatal and consequently on cortical transmission occur mostly by somatodendritic release (Gonon, 1997; Cragg and Rice, 2004; Rice and Cragg, 2004; see Figure 1). Geffen et al. (1976) suggested that the process of somatodendritic dopaminergic release is vesicular and exocytotic, like axonal release (see Figure 1). The release of a single vesicle of dopamine could modulate the excitability of tens to thousands of synapses within a few micrometers of a release site, in both substantia nigra compacta and striatum (Cragg et al., 2001; Rice and Cragg, 2008). It has been proposed that the sphere-of-influence of dopamine spillover in a concentration sufficient to stimulate dopamine receptors, has a radius of 2–8 μm (Rice and Cragg, 2008; see also Gonon, 1997; Cragg and Rice, 2004; Arbuthnott and Wickens, 2007). In addition, there is evidence for a lack of post-synaptic specialization at 60–70% of suggested dopamine release sites in the striatum (Descarries et al., 1996) and limited evidence for either pre- or post-synaptic specializations to delineate somatodendritic dopamine release sites in midbrain dopaminergic neurons (Wilson et al., 1977). Spillover is required for signaling by dopamine since its receptors are largely extrasynaptic (Cragg et al., 2004). This extrasynaptic interaction would be a communication intermediary between classic neurotransmission and the relatively non-specific neuroendocrine secretion (Agnati et al., 1986, 1995, 2010; Descarries et al., 1996; Descarries and Mechawar, 2000; Cragg and Rice, 2004; Bullock et al., 2005; Fuxe et al., 2007, 2010; De-Miguel and Fuxe, 2012).

Neurochemical and morphological evidence indicate that many other neurotransmitters may be released from both synaptic and extrasynaptic sites for diffusion to target cells (Agnati et al., 1986, 1995, 2010; Descarries et al., 1996; Descarries and Mechawar, 2000; Cragg and Rice, 2004; Bullock et al., 2005; De-Miguel and Trueta, 2005; Fuxe et al., 2007, 2010; De-Miguel and Fuxe, 2012). Dopaminergic signals can be inhibited or enhanced by a variety of identified factors that regulate axonal and somatodendritic dopamine release. Specific factors include dopamine autoreceptors, Ca^2+^, glutamate, GABA, acethilcholine, opioids, cannabinoids, and the diffusible messengers hydrogen peroxide and nitric oxide (Wightman, 2006; Rice et al., 2011). Recent findings indicate that nitric oxide may be involved in the extrasynaptic interactions (Figure 1; Kiss and Vizi, 2001; Kiss et al., 2004; Steinert et al., 2008). Volume signaling by nitric oxide is supported by studies modeling the spread of nitric oxide in space and time (Philippides et al., 2000). The function of nitric oxide as a “volume” transmitter has been proposed in systems where the source and target of nitric oxide are anatomically segregated (Gally et al., 1990; Wood and Garthwaite, 1994; Philippides et al., 2005; Steinert et al., 2008). Artinian et al. (2010) support the notion that cellular nitric oxide release can modulate neuronal firing properties in synaptically unconnected neurons, in direct support of nitric oxide functioning as a volume messenger.

Nitric oxide is a gaseous neurotransmitter and an unstable free radical highly diffusible in aqueous and lipid environments. Nitric oxide is produced from l-Arginine by nitric oxide synthase (NOS) after *N*-Methyl d-Aspartate (NMDA) receptor activation and calcium influx (Snyder and Ferris, 2000; Garthwaite, 2008). Once inside target cells, nitric oxide binds the iron in the heme group contained within the active site of soluble guanylyl cyclase, thereby activating the enzyme to form cyclic guanosine monophosphate and other effector enzymes located in pre-synaptic and post-synaptic elements (Garthwaite and Boulton, 1995; Garthwaite, 2008). Also, nitric oxide can serve as a link between monoaminergic and glutamatergic synaptic extrasynaptic transmission (Kiss and Vizi, 2001; Vizi et al., 2004).

Given that nitric oxide is freely diffusible and thus can readily enter adjacent neuronal cells or other cells, NOS enzymatic activity is exceptionally regulated (Snyder and Ferris, 2000). There are at least three different forms of this enzyme (Griffith and Stuehr, 1995): the endothelial that is responsible essentially for cardiovascular actions, the inducible form found originally in macrophages and involved mainly in immune processes and the neuronal NOS (nNOS), present mostly in neurons but also in mitochondria and chondrocytes. All NOS contain multiple regulation sites (Griffith and Stuehr, 1995; see also Garthwaite, 2008; Steinert et al., 2008 for review). Even though nearly all oxidative enzymes employ one electron donor, NOS is further complexed (Snyder and Ferris, 2000) since it possesses a tightly bound flavin adenine mononucleotide and dinucleotide, in addition to NADPH. It further utilizes heme and tetrahydrobiopterin as electron donors. NOS possesses sites for phosphorylation by the major phosphorylating enzymes, including cyclic AMP-dependent protein kinase, protein kinase C, calcium-calmodulin-dependent protein kinase, and cyclic guanosine monophosphate-dependent protein kinase (see Garthwaite, 2008; Steinert et al., 2008 for review). Hence, NOS must be activated every time a neuron releases nitric oxide, which is then released as soon as it is synthesized.

Neuronal NOS is a cytoplasmic enzyme (Garthwaite, 2008) that is expressed by discreet populations of neurons in the central nervous system (Vincent and Kimura, 1992). All nNOS interneurons express also nicotinamide adenine dinucleotide phosphate diaphorase (NADPH-d; Sancesario et al., 2004; see also Rushlow et al., 1995; Figueredo-Cardenas et al., 1996). Within the striatum, interneurons expressing nNOS are well characterized by cytochemical and physiological criteria (Kawaguchi, 1993; Kawaguchi et al., 1995, 1997). nNOS-containing interneurons represent only about 1% of neuronal cells in the brain (Sancesario et al., 2004; see also Rushlow et al., 1995; Figueredo-Cardenas et al., 1996). Because nNOS is not necessarily confined to pre- or post-synaptic specializations, nitric oxide can potentially be released from extensive extrasynaptic regions of nitrergic neurons (Philippides et al., 2005). Therefore, the somata and dendrites of nNOS cells represent potentially large sources of nitric oxide (see Figure 1).

Nitric oxide is an activity-dependent volume modulator, adapting intrinsic excitability and synaptic efficacy and modulating both active and inactive neuronal populations to the same physiological input (Gally et al., 1990; Philippides et al., 2005; Steinert et al., 2008). The proximity of the target cells is one factor that will determine the strength of the nitric oxide signal they receive. Even if the signal from a single small nitric oxide source is very local, multiple active sources can cooperate to cover large tissue volumes with nitric oxide concentrations that exceed those generated at the surface of a singly active source (Philippides et al., 2005). The proximity of the nitrergic inputs to dopaminergic inputs on medium spiny neurons in the striatum (Hidaka and Totterdell, 2001) suggests an important role for nitric oxide transmission in modulating the responsiveness of medium spiny neurons to afferent drive (for review see West et al., 2002). This consideration raises the possibility that the topography of nNOS-containing cells and the degree of their overlap with dopaminergic neurons may be of functional significance.

Dopamine neurons are easily identified by the presence of TH(+), a rate-limiting enzyme for catecholamine synthesis that catalyzes the conversion of tyrosine to l-DOPA, the first step in catecholamine neurotransmitter production (Hökfelt et al., 1976, 1977). TH(+) immunoreactive fibers and neurons are generally regarded as dopaminergic (Bouyer et al., 1984). In the brain, the synthetic enzyme for nitric oxide in neurons, the nNOS, is easily identified (Kawaguchi, 1993; Rushlow et al., 1995; Figueredo-Cardenas et al., 1996). Abundant evidence indicates interaction between nitrergic and dopaminergic systems playing an important role in the control of motor function and in the context of neurodegenerative diseases, such as Parkinson’s (Galati et al., 2008; Gomes et al., 2008) and l-DOPA-induced dyskinesia (Padovan-Neto et al., 2009, 2011; Novaretti et al., 2010; Yuste et al., 2011; Takuma et al., 2012; for review see Jenner, 2008; Del Bel et al., 2011; Iravani and Jenner, 2011; Pierucci et al., 2011; West and Tseng, 2011; Iravani et al., 2012). A similar localization/interaction in either neuronal compartment would reinforce the suggestion, whereby nitric oxide influences the outcome of a movement, perhaps, opening new strategies for novel therapeutic regimes.

Our aim is to explore the hypothesis that the spatial relationships between either axons or somata of the nitric oxide and dopaminergic cells may represent selective associations between the nitrergic and dopaminergic systems. For this purpose, the dual localization of immunoreactivity for NOS and tyrosine hydroxylase in neurons of the nigrostriatal pathway in the rat brain was examined by means of a double-immunohistochemical method and confocal laser scanning microscopy (CLSM).

## Materials and Methods

### Animals

Adult male *Wistar* rats (200–250 g) were housed in groups of five per cage in a temperature-controlled room (23°C), under 12 h light-dark cycles with free access to food and water. Experiments were conducted according to the principles and procedures described by the Guidelines for the Care and Use of Mammals in Neuroscience and Behavioral Research (ILAR, USA) and Guidelines from the School of Medicine (USP, Brazil). The institutions housing conditions and experimental procedures were previously approved by the local Animal Ethics Committee.

### Tissue processing

Rats were deeply anesthetized with urethane (25 mg/kg) and then rapidly perfused transcardially with 250 ml of cold saline and 400 ml of 4% paraformaldehyde (PFA) in 0.1 M phosphate buffer (pH 7.4). Brains were immediately removed and fixed in 4% PFA for 24 h and cryoprotected in 30% sucrose solution. Brains were snap frozen in isopenthane (−40°C, Sigma) and stored at −70°C till use. The tissues were cut at 40 μm on a cryostat. Sections through the brain regions were collected in 0.01 M PBS solution containing 0.02% sodium azide and stored at 4°C until use.

### Immunohistochemistry reaction

Herbison et al. (1996) reported the production and specificity of the K205 sheep anti-rat nNOS antibody and, in particular, noted that it detects one main protein with a molecular mass of 155 kDa in the rat brain. To assess antibody specificity within the rat brainstem, liquid phase adsorption experiments were undertaken in the present study by incubating the K205 at working dilution (1:5000) with recombinant rat nNOS (1 mM) overnight at 4°C and then carrying out immunocytochemistry on brainstem sections using the adsorbed antiserum in parallel with the normal K205 (Simonian and Herbison, 1996). These experiments showed that all immunoreactivity was abolished by adsorption of the K205 antiserum.

Immunohistochemistry was performed using a standard peroxidase-based method (Gomes et al., 2008). The sections were incubated with the TH-specific primary antibody (1:2000, Pel Freez, Rogers, AR, USA) overnight at 4°C, followed by biotinylated secondary antibody (Vectastain ABC kit, Vector Laboratories) and HRP-conjugated streptavidin (Vectastain ABC kit, Vector laboratories) incubation. The sections were developed using diaminobenzidine as the chromogen, mounted on slides and cover slipped for subsequent microscopic observation. Structure localization was determined with the help of the Paxinos and Watson (1999). Images containing TH immunostaining were captured with a digital Olympus DP70 camera mounted on a wide field microscope. Immunoreactive cells in a field of 1.02 mm × 0.78 mm were then counted using the image processing and analysis software package Image J.

Double-immunolabeling was carried out in the same brain region to characterize nNOS and TH expression. The sections were incubated overnight at 4°C with an anti-TH antibody (1:1000, Pel Freez, Rogers, AR, USA) followed by a 2 h incubation at room temperature with a secondary donkey anti-mouse IgG (H + L) antibody coupled to Alexa Fluor 488 (1:250; Invitrogen, Scotland, UK). After TH labeling, the tissue was washed three times with PBS and incubated overnight with a polyclonal antibody specific for rat nNOS (1:1000; sheep anti-nNOS, K205, gift of P. Emson, Cambridge, UK). The primary nNOS antibody was then revealed with a Cy3-coupled donkey anti-sheep IgG (H + L; 1:250; Jackson Immuno Research, USA). The primary antibodies were diluted in PBS (0.1 M; pH 7.4) containing 0.02% thimerosal, 5% normal goat serum (NGS, Jackson Immuno Research, USA), 0.2% Triton X-100, while the secondary antibodies were diluted only in PBS (0.1 M; pH 7.4).

Controls for the fluorescent double-labeling experiments involved the omission of one of the primary antibodies in the presence of the other and both secondary antibodies. In these experiments, neither Cy3 nor Alexa Fluor 488 staining was apparent on cells when either the TH or the nNOS antiserum was omitted. Double-stained sections were analyzed using a fluorescence microscopy setup (Nikon, Japan) equipped with a 60x objective (numerical aperture 1.4) and connected to an image analysis system (Mercator, Explora Nova, La Rochelle, France). A total of six sections of each selected brain region, encompassing rostral, middle, and caudal levels of the striatum, subthalamic nuclei, substantia nigra *pars* compacta and reticulate, ventral tegmental area, and pedunculopontine nuclei were examined, as previously described (Debeir et al., 2005).

### Cell nuclei labeling

A fluorescent stain that labels DNA and allows for easy visualization of the nucleus in interphase cells and chromosomes in mitotic cells is 4′,6-diamidino-2-phenylindole (DAPI, Chazotte, 2011). DAPI (10 mg/mL in H_2_O stock solution; Invitrogen D1306) was diluted 1:5000 with PBS (DAPI labeling solution). The slides were incubated for 1–5 min at room temperature in DAPI labeling solution. The slides were rinsed three times in PBS^+^ and the coverslips mounted (Chazotte, 2011).

### Confocal laser scanning microscopy

Sections double-stained for nNOS and TH were inspected using a Leica SP2 and Leica SP5 confocal laser scanning microscopes (both Leica Microsystems, Wetzlar, Germany). Z-stacks of the frontal cortex were acquired with a 40× oil objective (1.25NA, HCX PL APO CS), while regions from the striatum and substantia nigra were acquired at the optical resolution limit (43 nm× 43 nm × 130 nm) with a 63× (HCX PL APO) oil objective and subsequently deconvolved with AutoQuant^®^X (Ver. X2.2.1, Media Cybernetics, MD, USA) to obtain an improved signal-to-noise ratio. The Alexa Fluor 488 fluorophore (Abs = 495 nm, Em = 519 nm) was excited with the 488 nm laser line of an Ar-laser, while the Cy3 fluorophore (Abs = 550 nm, Em = 570 nm) was excited at 561 nm, with emission being collected in a line-by-line fashion at 500–530 and 570–650 nm, respectively.

### Tyrosine hydroxylase and nitric oxide synthase-positive structures proximity analysis

**Figure 2:**
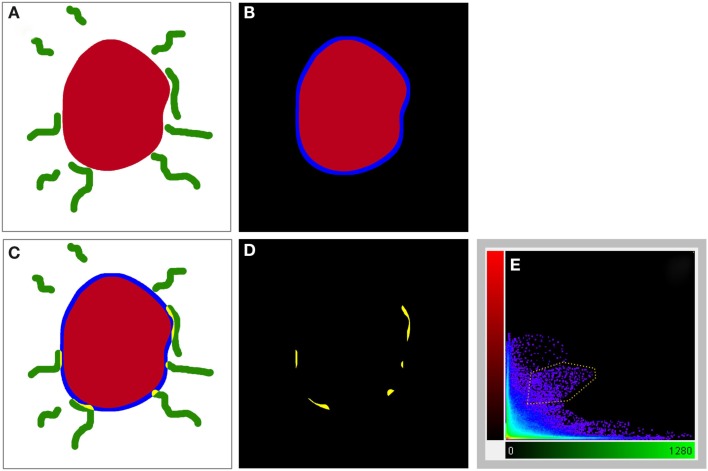
**A schematic illustrating the method used to visualize processes present in the cell periphery**. The cell body (red) was thresholded **(A)** to generate a binary mask and then enlarged [blue line in **(B,C)**] to occupy an additional 2 μm around its periphery. Areas occupied within the blue periphery by the green processes are shown in [**(C,D)**; yellow areas]. In **(D)**, only the pixels where processes are within the 2 μm proximity of the cell body are shown. A 2D scatter plot **(E)** was generated using Imaris 7.5 (Bitplane) revealing a pixel population (outlined with yellow line) with correlating intensities (Pearson ∼0.7), out of which a new channel was generated.

For the ensuing analysis, cells were chosen whose the major, longitudinal axis is orthogonal with respect to the *z*-axis of the stack. A maximum intensity projection was then generated with Fiji software package (Walter et al., 2010) from the nNOS(+) and TH(+) channels, respectively, based on a 5 μm-thick optical section centered at the widest *z*-position of the cell body. Binary masks were then generated from both maximum projection types (schematic representation in Figure 2A). Fiji was then used to grow the binary mask of the cell body such that the resulting area covered an additional 2 μm-wide margin at the periphery of the nNOS(+) immunoreactive cell body (blue margin in Figure 2B). The expanded binary mask depicting the cell body was added to the mask representing the fibrous structures (Figure 2C). In a final step, pixels within this newly generated margin that were positive for both, nNOS(+) and TH(+) immunoreactivity were identified (yellow pixels in Figures 2C,D). Imaris 7.4.2 (Bitplane) was used to generate 3D representations of the respective z-stacks and for colocalization analysis (Figure 2E).

## Results

Both hemispheres of all animals showed a similar distribution pattern of the TH(+) and nNOS(+) immunoreactive cells and fibers within the striatum-nigra pathway. Using CLSM optical sections acquired at the resolution limit, colocalized regions (Pearson correlation coefficient ≥0.7) were identified (yellow). The yellow pseudo-color for these structures visible in CLSM images could represent the superimposition of TH(+) immunoreactivity (green) on nNOS(+) immunoreactivity (red) neurons/fibers and *vice versa*. Assessment of sections double-stained for nNOS(+) and TH(+) immunoreactivity revealed a considerable overlap of their topographies. The distribution of nNOS(+) immunoreactivity is generally in agreement with the data published by others using either an immunocytochemical (Bredt et al., 1990; Schmidt et al., 1992; Egberongbe et al., 1994; DeVente et al., 1998) or a histochemical approach (Vincent and Kimura, 1992; Southam and Garthwaite, 1993).

### Cortex

The prefrontal, anterior cingulate, insular, piriform, perirhinal, entorhinal, motor, premotor, parietal, temporal, and posterior cingulate cortices had a dense distribution of TH(+) immunoreactive fibers. A small population of medium-large, intensely nNOS(+) immunoreactive multipolar neurons was found scattered throughout layers 2–4 of all examined cortices. The reaction product for nNOS(+) immunohistochemistry in the cortical neurons formed dense, conspicuous masses with no nuclei labeling.

**Figure 3:**
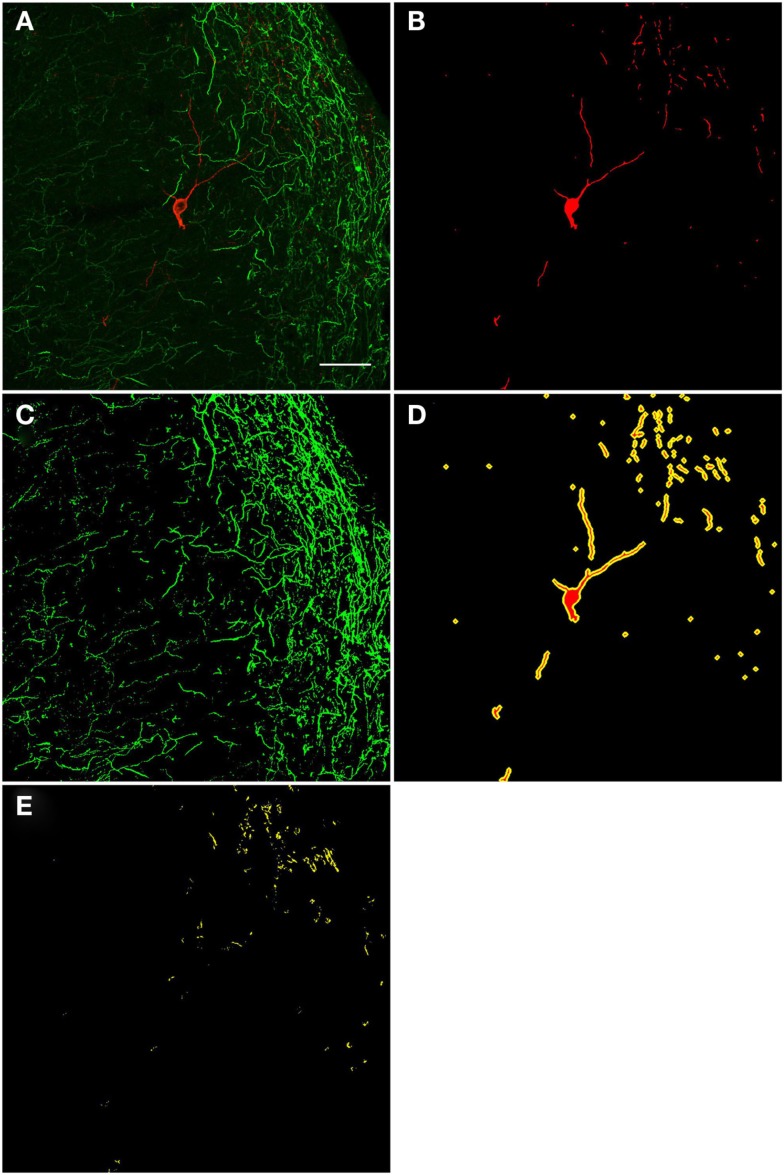
**Confocal laser scanning microscopy of TH(+) and nNOS(+) double-immunostained sections from the rat cingulate cortex**. The TH(+) immunoreactivity **(A,C)** is shown in green, whereas the nNOS(+) immunoreactivity **(A,B)** is red, in the same double-stained section. In **(B,C)**, the binary masks of the nNOS(+) and TH(+) signals are shown, respectively, while **(D)** additionally shows the 2 μm-wide periphery (yellow) of the nNOS(+) cell body and processes (red). The yellow areas in **(E)** indicate the location of TH(+) processes within the 2 μm periphery of the nNOS(+) cell. There is absence of complete colocalization between nNOS(+) and TH(+) immunoreactivity in the rat cortex but a large number of fibers are present within the 2 μm nNOS(+) cell periphery. Scale bar is 50 μm.

A qualitative analysis of the area 2 μm proximal to the nNOS(+) cell body was carried out (Figure 3) in the anterior cingulate cortex (Bregma 0.797–0.251, Paxinos and Watson, 2009). The TH(+) immunoreactivity (Figures 3A,C) is shown in green, whereas the nNOS(+) immunoreactivity (Figures 3A,B) is red, in the same double-stained section. In Figures 3B,C, the binary masks of the nNOS(+) and TH(+) signals are shown, respectively. The yellow area in Figure 3D represents the 2 μm periphery of the nNOS(+) cell. Figure 3E shows only areas where the TH(+) immunoreactive cell body or processes are located within the 2 μm proximity of the nNOS(+) immunoreactive cell body or processes thereof.

### Striatum

**Figure 4:**
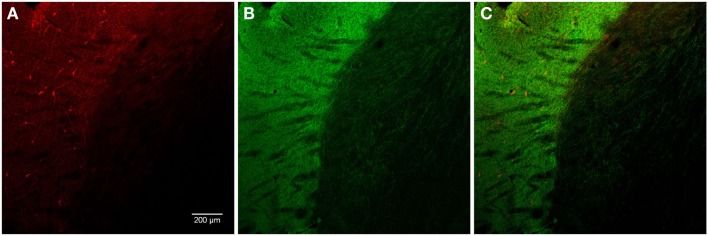
**Photomicrographs of nNOS(+) immunoreactive neurons (A) and TH(+) nerve fibers (B) in sections from the caudate–putamen/globus pallidus [merge in (C)]**. nNOS(+) immunoreactive neurons appear polygonal or fusiform in shape with long smooth dendrites and a plexus of nerve fibers in the neuropil. The low-power photomicrograph shows the intense staining of the TH(+) immunoreactivity and the large number of nNOS(+) cell body in the caudal striatum. Scale bar = 200 μm.

In general, nNOS(+) immunolabeling was detected in the cytoplasmic domains of the cell body and dendrites. The nucleus was unstained and the reaction product was evenly distributed in the cytoplasm. A dense network of NOS(+) varicose fibers was observed in the striatal complex. TH(+) immunohistochemistry demonstrated TH(+) immunoreactive terminals or varicose fibers in the neuropil of the striatum (Figures 4, 6C, and 8D).

**Figure 5:**
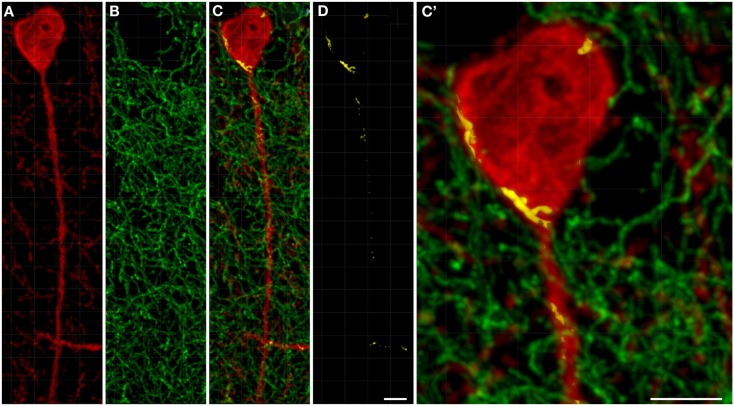
**Confocal laser scanning microscopy of TH(+) and nNOS(+) immunoreactive double-immunostained sections from the rat striatum**. Fibers immunoreactive for TH(+; green) are presented in **(B,C,C′)**. A neuron immunoreactive for nNOS(+)(red) is presented in **(A,C,C′)**. Using CLSM optical sections acquired at the resolution limit, colocalized regions (Pearson correlation coefficient of ≥0.7) were identified (yellow). The yellow areas in **(C,D,C′)** also indicate the location of TH(+) processes within the 2 μm periphery of the nNOS(+) cell. Detail of the cell body is presented in **(C′)**. In the rat striatum a large number of TH(+) fibers are present within the 2 μm nNOS(+) cell body. Scale bar in **(D,C′)** = 5 μm.

**Figure 6:**
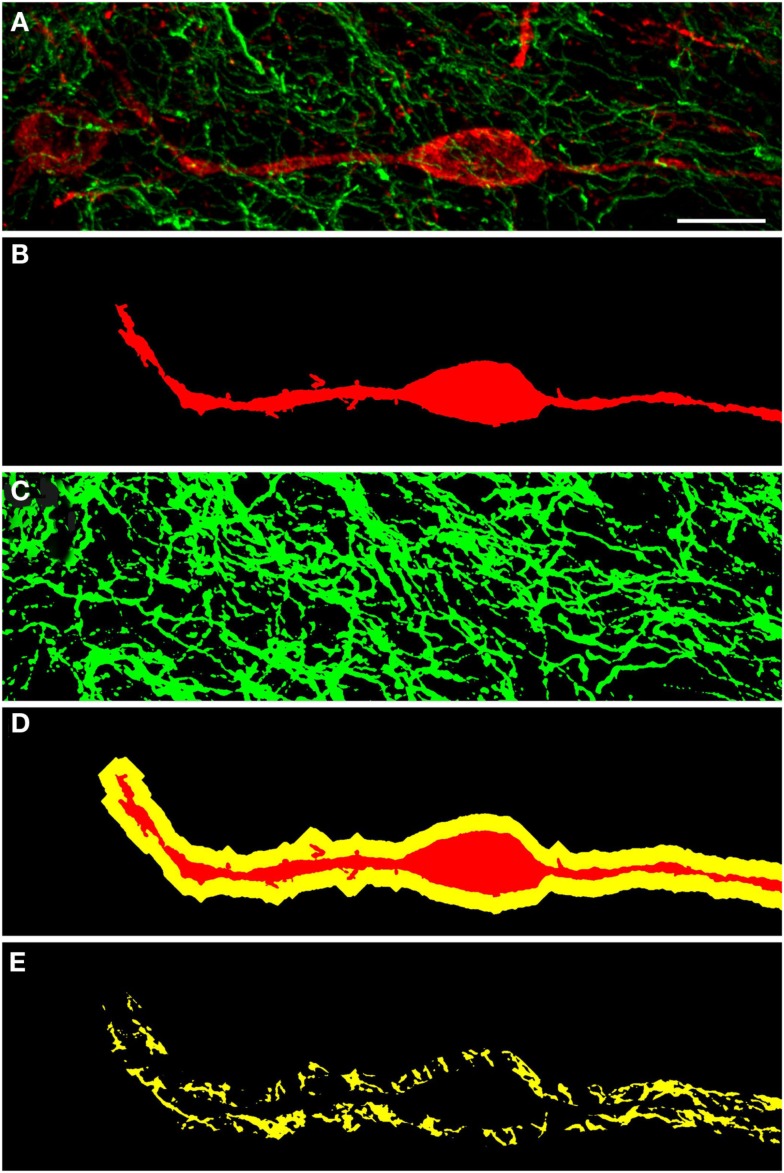
**Confocal laser scanning microscopy of TH(+) and nNOS(+) immunoreactive double-immunostained sections from the rat striatum: a proximity analysis study**. Distribution pattern of immunoreactivity for TH(+) and a nNOS(+) in double-stained sections of the striatum. Both, nNOS (red) and TH (green) fluorescent signals are shown in **(A)**, while **(B,C)** show the corresponding binary masks. The 2 μm periphery (yellow) is shown in **(D)** and the area occupied by TH(+) immunoreactive fibers around the nNOS(+) immunoreactive cell body is shown in **(E)**. The yellow areas in E indicate the location of TH(+) and nNOS(+) possible interaction. Scale bar in **(A)** is 10 μm.

TH(+) immunolabeling in the striatum appeared as a dense, inhomogeneous plexus of fibers with an absence of cell bodies at any level (see Moss and Bolam, 2008). Several punctate structures (Figures 5C,C′,D and 6D,E; yellow dots) showing TH(+) immunoreactivity and nNOS(+) were in close contact. They were surrounding either the cell bodies or the cell processes (see Figures 5 and 6). The yellow areas in Figures 5C,D,C′ also indicate the location of TH(+) processes within the 2 μm periphery of the nNOS(+) cell. Detail of the cell body is presented in Figure 5C′.

**Figure 7:**
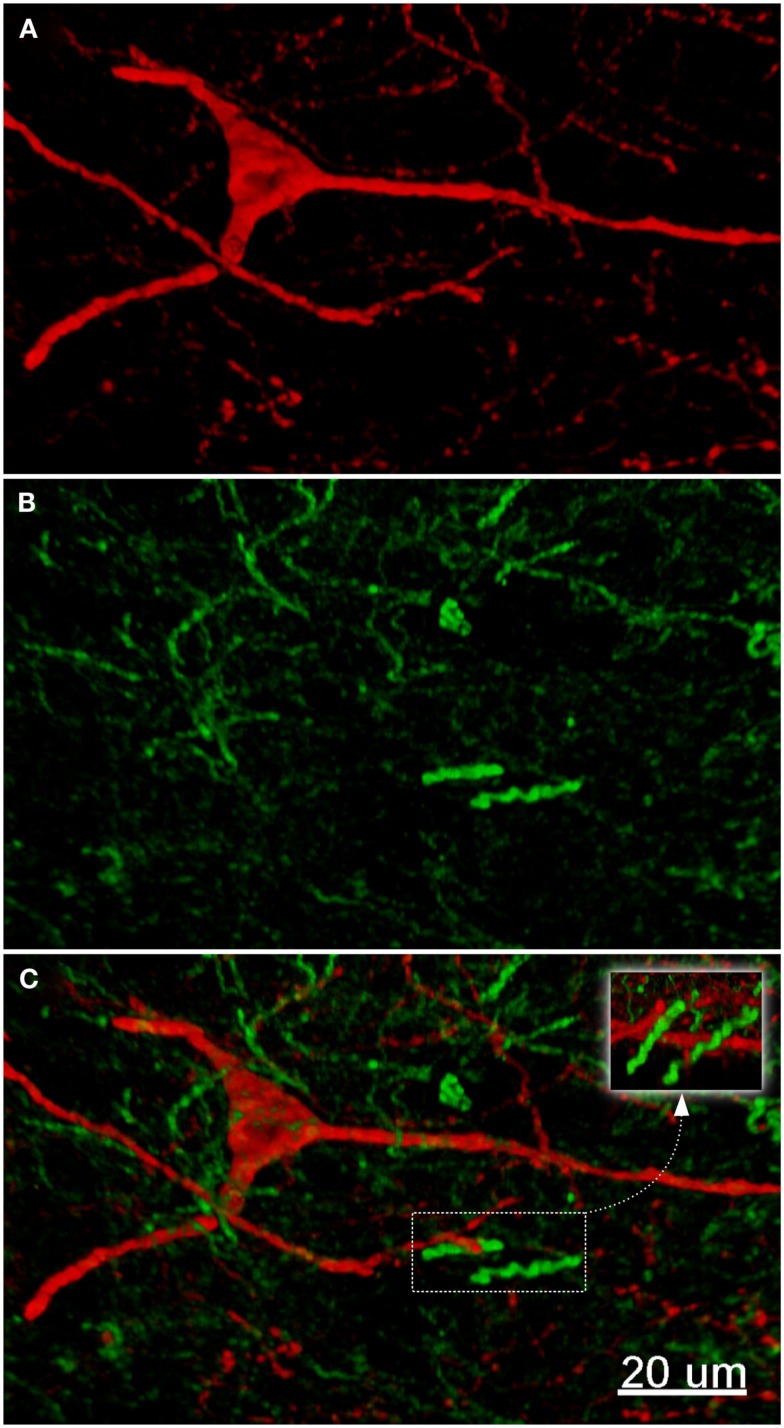
**The fluorescence pattern in a double-stained section of the nucleus accumbens, showing nNOS(+) [(A,C) – cell body) and TH(+) [(B,C) axons] immunoreactivities**. Immunoreactivity for TH(+) and a nNOS(+) in double-stained sections of the nucleus accumbens. Both, nNOS (red) and TH (green) fluorescent signals are shown in **(C)**. Whereas the TH(+) fiber immunoreactivity appears homogeneously distributed throughout the whole nuclei, the nNOS(+) immunoreactive neurons and fibers are stained much more sparsely. Scale bar in **(A)** = 10 μm, it applies to **(B,C)** also.

The nucleus accumbens (ventral striatum) displayed TH(+) immunoreactivity and nNOS(+) immunoreactivity similar to that of the dorsal striatum (Figure 7). There was a range of nNOS(+) cell body shapes, from which two to four smooth and extensive, poorly branching dendrites emanated, similar to those described by Hussain et al. (1996). The nNOS(+) immunoreactive cells were of medium size, fusiform and spiny interneurons with long, poorly branched dendrites that comprise only a small percentage of the striatal neuronal population.

### Globus pallidus external and internal (entopeduncular nuclei)

**Figure 8:**
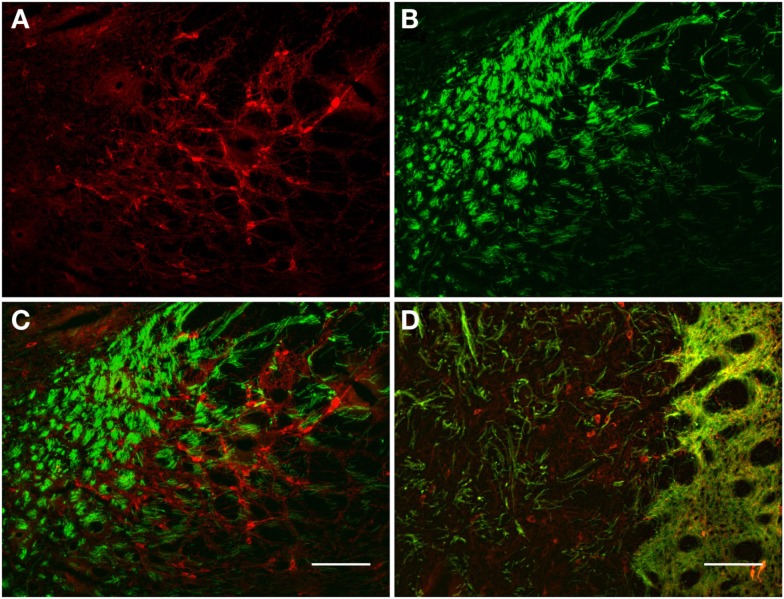
**Internal globus pallidus [entopeduncular nucleus-(A,B), merge in (C)] and external globus pallidus (D) photomicrographs of nNOS(+) immunoreactive neurons and TH(+) nerve fibers**. **(A–C)** TH(+) and nNOS(+) immunoreactive fiber staining representative of the typical honeycomb-like pattern in the entopeduncular nucleus [merge in **(C)**]. TH(+) immunoreactivity corresponds to the medial forebrain bundle. **(D)** Photomicrography of nNOS(+) immunoreactive neurons and TH(+) nerve fibers (merge) from a section of the external globus pallidus/caudal striatum. Note the nNOS(+) immunoreactive cells (red), the dark staining surrounds areas of lighter immunoreactivity (external globus pallidus) and the dense TH(+) fibers in the striatum. nNOS(+) immunoreactive neurons appear polygonal in shape with long smooth dendrites and a plexus of nerve fibers in the neuropil. Scale bars, 100 μm.

**Figure 9:**
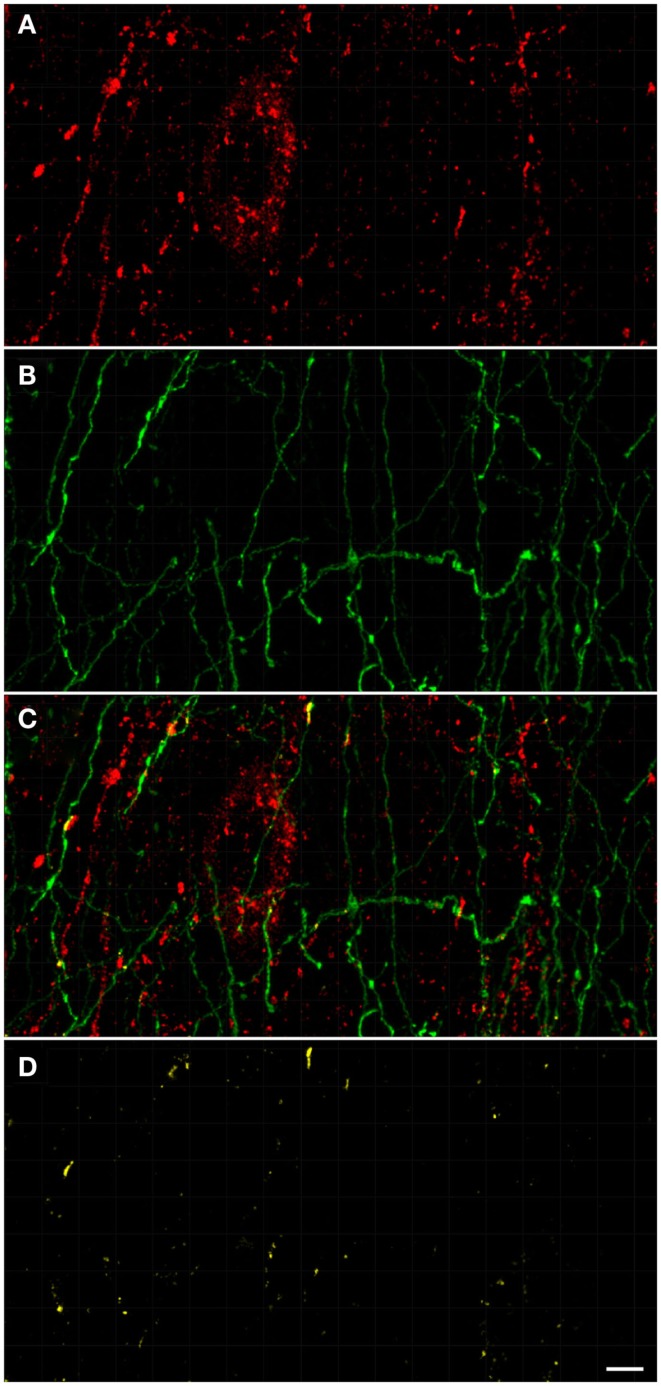
**Confocal laser scanning microscopy of the external globus pallidus in TH(+) and nNOS(+) immunoreactive double-immunostained sections**. The axons are immunoreactive for TH(+) [green, **(B,C)**] and the neurons/axons immunoreactive for NOS(+) [**(A,C)**, red] in double-stained sections of the external globus pallidus. Both, TH(+) and nNOS(+) immunoreactivities are presented in **(C)**. Observe the lower fluorescence of nNOS(+) immunoreactive cells surrounded by areas of lighter immunoreactivity of the TH(+)fibers. The nNOS(+) immunoreactivity delineating a neuronal cell body structure is covered by dots of TH(+) immunolabeling in yellow **(C,D)**. Using CLSM, zones of cellular colocalization were ascertained [yellow, **(C,D)**]. Scale bar is 25 μm.

The TH(+) and nNOS(+) immunoreactivity double-labeling in the pallidus external and internal (entopeduncular nucleus in the rat) is presented in Figures 8 and 9. In the internal globus pallidus we observed clusters of numerous NOS(+) cell bodies. TH(+) and nNOS(+) immunoreactivity in the fiber staining represent a typical honeycomb-like pattern (Figure 8). TH(+) immunoreactivity corresponds to the medial forebrain bundle. In Figure 8D, note the nNOS(+) immunoreactive cells (red) in the external globus pallidus and the less intense staining surrounding areas of lighter immunoreactivity. nNOS(+) immunoreactive neurons appear polygonal in shape with long smooth dendrites and a plexus of nerve fibers in the neuropil.

In the external globus pallidus (Figures 8D and 9), there is a distinct punctuate pericellular nNOS(+) labeling of the whole neuronal soma and dendritic/axonal processes (Figure 9). TH(+) fibers make multiple contacts onto the nNOS(+) immunoreactive delineated structure. Using CLSM, zones of cellular colocalization were ascertained (yellow, Figures 9C,D).

### Substantia nigra/ventral tegmental area

**Figure 10:**
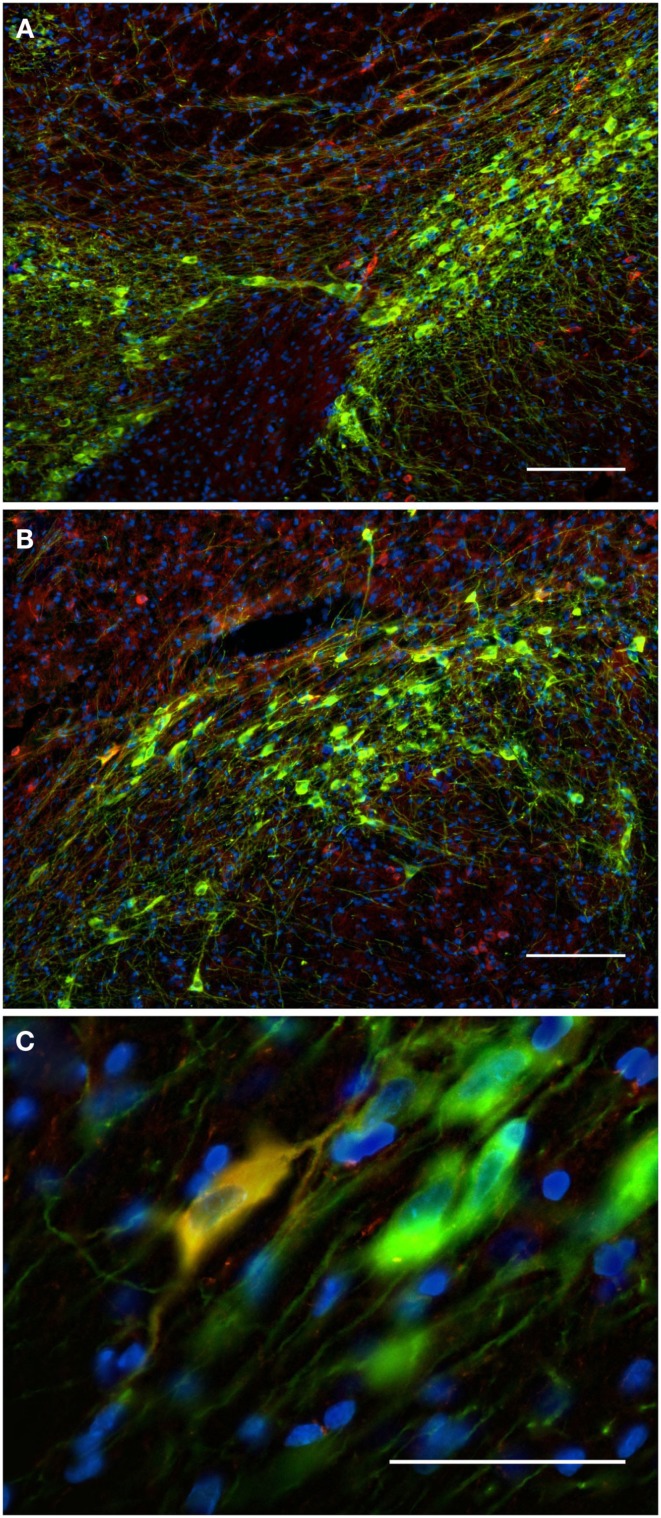
**The substantia nigra compacta and ventral tegmental area nNOS(+) and TH(+) immunoreactive neurons and fibers**. Distribution pattern of axons/cells immunoreactive for nNOS(+) **(A)** and TH(+) **(B)** in double-stained sections of the substantia nigra [**(C)**, merge]. Cell nuclei are labeled in blue with DAPI. Using CLSM **(A–C)**, zones of cellular colocalization were demonstrated in a single optical section of the substantia nigra compacta [**(C)**, scale bar is = 5 μm]. Observe the dense staining of the TH(+) immunoreactive cells and fibers homogeneously distributed throughout the substantia nigra compacta and the ventral tegmental area **(A)**. In the substantia nigra, dopaminergic cells are arranged in two bands (Fallon and Loughlin, 1995). The substantia nigra reticulate caudoventral emits cell bridges that make contact with the substantia nigra compacta rostrodorsal [González-Hernández and Rodríguez, 2000; **(A,B)**]. The substantia nigra compacta is characterized by a high density of dopaminergic somata as well as a dense network of overlapping dopaminergic dendrites. Scattered neurons in both substantia nigra compacta and reticulata display immunoreactivity to nNOS [Rodrigo et al., 1994; **(A,B)**]. Cell colocalization between nNOS(+) and TH (+) immunoreactivity is almost absent but a double-labeled cell is visualized in **(C)**. Scale bars, 50 μm **(A–C)**.

Most TH(+) immunoreactivity is grouped in the substantia nigra *pars* compacta, where it is spread forming a dense band that lies dorsally from the ventral tegmental area (see Figure 10). In the substantia nigra *pars* reticulata particularly in its medial two-thirds, a few TH(+) immunoreactive cells are present, some of them arranged in linear bridges which cross the substantia nigra *pars* reticulata perpendicularly to the substantia nigra *pars* compacta (see Figure 10B). nNOS(+) immunoreactive neurons appear occasionally in the substantia nigra *pars* compacta, stained much more sparsely, with long smooth dendrites (Figures 10A,B). In the substantia nigra *pars* compacta we found a dense pattern of TH(+) immunoreactive cells and fibers next to a small number of fibers and cell somata with strong NOS(+) immunoreactivity (Figures 10A,B). A double-labeled cell is visualized in Figure 10C but cell colocalization between nNOS(+) and TH (+) immunoreactivity is almost absent.

**Figure 11:**
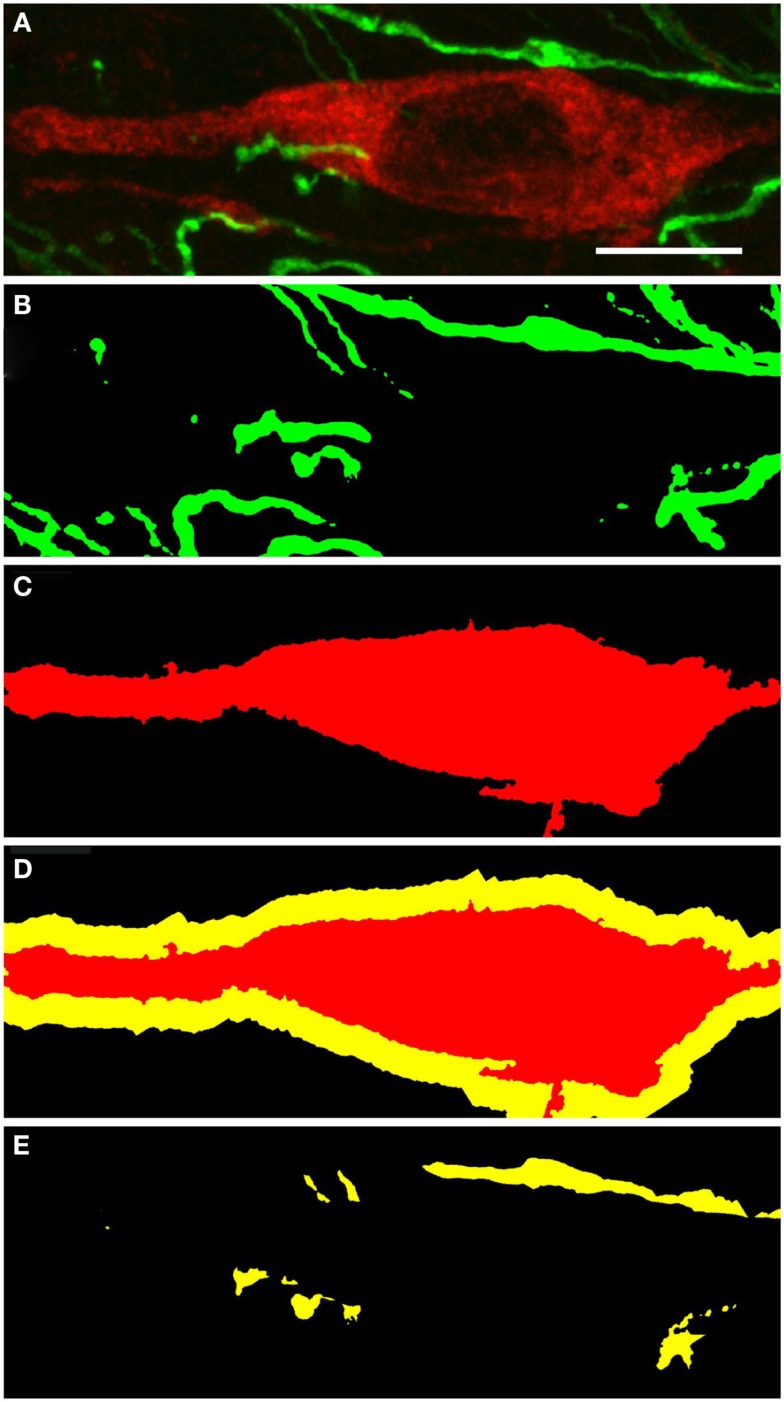
**Substantia nigra compacta nNOS(+) immunoreactive neuron and TH(+) fibers**. CLSM analysis of a double-immunostained section. **(A)** Show in detail a nNOS(+)immunoreactive neuron (red) surrounded by TH(+) immunoreactive fibers (green). **(B,C)** Show the binary masks of the TH(+) and nNOS(+) stains, respectively. In **(D)**, the 2 μm proximity of the nNOS(+) cell is shown, while in **(E)** the area occupied by the TH(+) processes within the 2 μm proximity is visualized. Zones of colocalization were demonstrated in a single optical section of the substantia nigra compacta acquired at the resolution limit. Scale bar 10 μm.

TH(+) immunoreactive and NOS(+) immunoreactive cells could be observed adjacent to each other, with areas of perfect match (Figure 11). Fibers and cell bodies are in close proximity (Figure 11). Figure 11 presents in further detail a nNOS(+) immunoreactive neuron surrounded by TH(+) immunoreactive fibers. The yellow area in Figure 11D represents the 2 μm periphery of the nNOS(+) neuron. In a further step, the area within this 2 μm periphery is visualized, where TH(+) processes are present (Figure 11E). Zones of colocalization were demonstrated in a single optical section of the substantia nigra compacta acquired at the resolution limit. Actual colocalization between the two markers was rarely observed. In Figure 11E the area occupied by the TH(+) processes within the 2 μm proximity is visualized.

### Subthalamic nucleus

**Figure 12:**
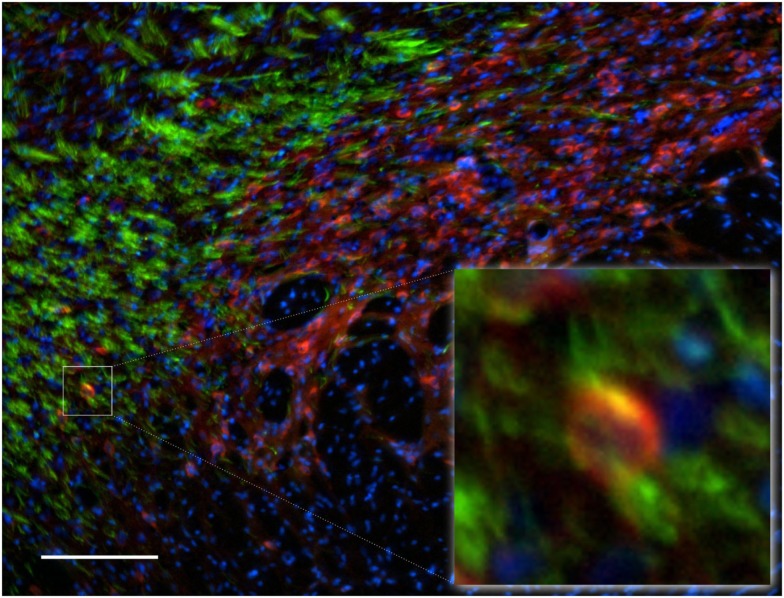
**Subthalamic nuclei of a control rat brain: distribution pattern of axons/cells immunoreactive for TH(+; green) and NOS(+; red) in double-stained sections**. The cell nuclei are labeled with DAPI. Observe the dense staining of the TH(+) immunoreactive fibers of the medial forebrain bundle (green fibers). nNOS(+) immunoreactive neurons appear massively at the subthalamic nuclei with long smooth dendrites. The area occupied by TH(+) immunoreactive fibers around the nNOS(+) immunoreactive cell body is shown in the inset. Cell colocalization between nNOS(+) and TH (+) immunoreactivity is absent. Scale bar is 100 μm.

Functional importance of the subthalamic nucleus neurons is reflected by its anatomical connections to the main output nuclei of the basal ganglia, including both segments of the globus pallidus and the substantia nigra. In the subthalamic nucleus we observed numerous NOS(+) immunoreactive cell bodies and an intense pattern in the TH(+) immunoreactive fiber staining (Figure 12), corresponding to the medial forebrain bundle. nNOS(+) immunoreactive neurons appear massively at the subthalamic nuclei with long smooth dendrites. A very small proportion of subthalamic nucleus neuronal somata were apposed by immunoreactive axons (Figure 12-insect). The cell nuclei are labeled with DAPI. Colocalization between the two markers was almost absent.

### Pedunculopontine tegmental nucleus

**Figure 13:**
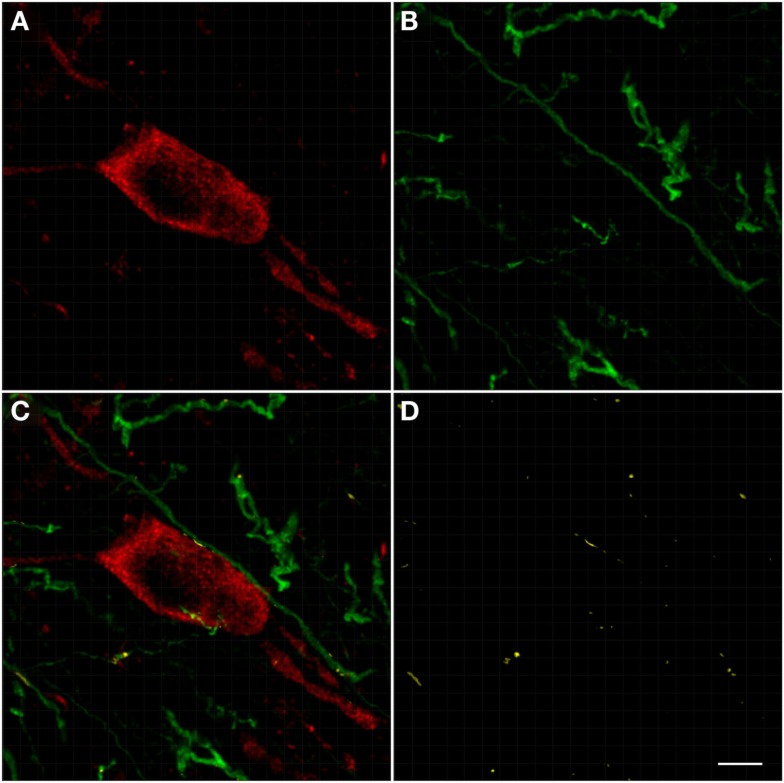
**Confocal laser scanning microscopy of the pedunculopontine nuclei in a TH(+) and nNOS(+) double-immunostained section**. Distribution pattern of axons/cells immunoreactive for TH(+) [green in **(B,C)**] and NOS(+) [red in **(A,C)**] is presented in this double-stained section. Observe the nNOS(+) immunoreactive neurons surrounded by sparse TH(+) fibers. TH(+) immunoreactive fibers appear scattered all around the cell soma. Scale bar 50 μm. Scale bar in **(D)** is 5 μm.

The pedunculopontine tegmental nuclei are a loosely defined aggregate of cholinergic and non-cholinergic neurons in the midbrain which neurons project to substantia nigra compacta dopaminergic neurons. Our observations revealed clusters of intensely labeled NOS(+) immunoreactive neurons (Figures 13A,C) and TH(+)immunoreactive fibers (Figures 13B,D) in the pedunculopontine tegmental nucleus (Figure 13). Double-labeling experiments indicate that the majority of TH(+) immunoreactive neurons in the pedunculopontine tegmental nucleus did not express NOS(+) immunoreactivity. However, fibers and cell bodies are in close proximity (Figure 13D).

## Discussion

We compared the spatial relationship between dopaminergic and nitrergic nigrostriatal fibers/cell body through the dual localization of the immunoreactivity for nNOS(+) and TH(+). The approach was based on double-immunohistochemical staining method using CLSM optical sections acquired at the resolution limit. A proximity analysis of TH(+) and nNOS(+) structures was done using binary masks generated from the respective maximum projections and revealed regions positive for both, nNOS(+) and TH(+) immunoreactivity, within a 2 μm-wide margin. Colocalized regions were identified with a Pearson correlation coefficient ≥0.7. A large proportion of nNOS(+) immunoreactive soma/axon/dendrite themselves were directly apposed by TH(+) immunoreactive ones, within a radius of 1 and 2 μm.

In the dopaminergic synapses, dopamine is released from pre-synaptic structures by exocytose, binds post-synaptic receptors and generates pre- and post-synaptic cascades of events (Bertorello et al., 1990; Beckstead et al., 2004; Jorgensen, 2004; Yao et al., 2008). The discovery that dopamine is located not only in the terminals but also in perikarya and dendrites of nigrostriatal neurons led to the suggestion that dopamine may be released from dendrites in the substantia nigra (Bjorklund and Lindvall, 1975; more information in the review from Trueta and De-Miguel, 2012). Subsequent immunohistochemical investigations have confirmed that the synthesizing enzymes (Tyrosine hydroxylase), for this catecholamine are also present in the dendrites of these neurons, some of which extend ventrally into the substantia nigra pars reticulata (Hökfelt et al., 1984; Jaeger et al., 1984). Release of dopamine from cell bodies and dendrites is typically referred to as somatodendritic release. Somatodendritic release of dopamine in the substantia nigra compacta and axonal dopamine release in the dorsal striatum are both necessary for the expression of basal ganglia-mediated motor behaviors and various cognitive functions. Dendritically released dopamine may modulate striatal outflow and thereby play an important role in basal ganglia function (Robertson and Robertson, 1987). Nigral dopamine neurons do not only release their transmitter into the striatum, but also interact with each other through release of dopamine from their somata and dendrites (Centonze et al., 2003; Rice and Cragg, 2004, 2008; complementary information in the review from Trueta and De-Miguel, 2012).

As described by Cragg and Rice (2004), the sphere-of-influence of dopamine spillover in a concentration adequate to stimulate dopamine receptor has a radius of 2–8 μm (see also Gonon, 1997; Arbuthnott and Wickens, 2007; Moss and Bolam, 2008; Rice and Cragg, 2008). Dopaminergic boutons correspond to almost 10% of all striatal synapses (Groves et al., 1994; Kreitzer, 2009), and the nearest-neighbor space among dopaminergic boutons is only 1.18 μm (Arbuthnott and Wickens, 2007; Kreitzer, 2009).

Nitric oxide at high concentrations can be used to mediate physiological responses in a restricted area over short periods (Kiss and Vizi, 2001; Kiss et al., 2004). It is predicted that the physiological volume of influence of a single source of nitric oxide that emits for 1–10 s has a diameter (in theory) of about 20 mm, corresponding to a volume of brain enclosing two million synapses (Wood and Garthwaite, 1994). Tornieri and Rehder (2007) demonstrated that nitric oxide has physiological effects at distances of up to 100 μm (Tornieri and Rehder, 2007). It was described by Ignarro (2010) that nNOS(+) immunoreactive neurons in the microenvironment could accomplish nitric oxide levels 100 times higher than required to stimulate for example, vasorelaxation of vascular smooth muscle cells. As described by Garthwaite and Boulton (1995), nitric oxide generated at a single point source should be able to influence function within a sphere with a diameter of 300–350 μm, even with a half-life of a few seconds, which is very large compared with the dimensions of a synapse. Taking into account some of the known radical scavenging properties of the tissues, Beckman and Koppenol (1996) reported the approximate ranges of diffusion for nitric oxide to be approximately 130 μm (Bidmon et al., 2001). Since medium-sized neurons are usually 10–25 μum in diameter, it becomes obvious that nitric oxide may diffuse into many cells

Therefore, our findings demonstrated that every nitric oxide molecule will be within overlapping spheres-of-influence of synaptically released dopamine in all the analyzed brain regions. Nitric oxide structures are likely to be within reach of a concentration of dopamine that is high enough to stimulate both high and low affinity receptors (Rice and Cragg, 2008). The topography of nNOS(+) and TH(+) immunoreactive cells and the degree of their overlap may be of functional significance.

Our observations are in agreement with Fujiyama and Masuko (1996); Simonian and Herbison (1996); DeVente et al. (2000); Hidaka and Totterdell (2001); Benavides-Piccione and DeFelipe (2003). In many cases, nitrergic terminals are closely apposed to TH(+) immunoreactive neuronal endings and both converge onto dendrites of spiny neurons (Hidaka and Totterdell, 2001). Alternatively, TH(+) immunoreactive neurons form direct synaptic contacts with nNOS(+) immunoreactive neurons (Hidaka and Totterdell, 2001). Fujiyama and Masuko (1996) simultaneously demonstrated NADPH-d reactivity and TH(+) immunoreactivity within the same section of the rat striatum using electron microscopy. Symmetrical and asymmetrical synaptic contacts were found on cell bodies and proximal dendrites. Other extrasynaptic TH(+) immunoreactive boutons were occasionally associated with unlabeled terminals adjacent to the NADPH-d positive dendrites (Fujiyama and Masuko, 1996). Double-labeling techniques showed that a small population of NOS(+) neurons in the medulla also contained immunoreactivity to the aminergic neuron marker TH (Dun et al., 1994). Also, occasionally double-labeling for nNOS(+) and TH(+) could be observed in some neurons in rat primary mesencephalic cultures (Salum et al., 2008). In contrast, Klejbor et al. (2004) found numerous TH(+) and NOS(+) immunoreactive neurons in the ventral tegmental area. The presence of nNOS(+) immunoreactive cell staining in the rat entopeduncular nucleus, with typical honeycomb-like pattern in the nucleus was hardly ever described before (Del Bel et al., 2011). Most frequently, in the external globus pallidus, the presence of nNOS(+) neurons was described as almost absent. However, in the external globus pallidus, we observed nNOS(+) immunoreactivity, with a distinct punctuate pericellular labeling which seems to correspond to the neuronal soma and/or dendritic/axonal processes. TH(+) fibers make multiple contacts onto the nNOS(+) immunoreactive delineated structure. It is curious that Arellano et al. (2004) and Castro et al. (2011) have shown similar pericellular pattern of parvalbumin-positive labeling cells in the hippocampus of epileptic patients.

These anatomical arrangements facilitate nitric oxide-mediated modulation of pre- and post-synaptic events such as dopamine release (Hanbauer et al., 1992; Sancesario et al., 2000; West and Grace, 2000; DiGiovanni et al., 2003; DiMatteo et al., 2009; Park and West, 2009). Studies showing that nNOS(+) immunoreactive interneurons facilitate the concurrent release of dopamine and glutamate by a nitric oxide-dependent process (West and Galloway, 1997; West and Grace, 2000; Park and West, 2009), suggest that nitric oxide might contribute in the integration of convergent motor information in striatal networks (West and Grace, 2000). As gap junction permeability between medium-sized, densely spiny neurons can be modulated by dopamine in the nucleus accumbens (O’Donnell and Grace, 1993), and by nitric oxide in the dorsal striatum (O’Donnell and Grace, 1997), these inputs may act together to modulate activities of individual projection neurons by means of conventional chemical synapses, or may influence electrotonic transmission between networks of neurons connected by gap junctions.

Consequently, even though we generally did not find soma colocalization in the analyzed regions, the implications of this finding are far-reaching. The ultimate distribution of dopaminergic and nitrergic structures in the nigro-striatal pathway is unlikely to be a targeted phenomenon, suggesting that the populations of TH(+) and NOS(+) immunoreactive neurons interact with each other in all analyzed regions. Together, our observations corroborate evidence of dopamine and nitric oxide being intertwined in the anatomy in addition to physiology and pathology of the nigrostriatal pathway (Del Bel et al., 2005, 2011; Jenner, 2008; Pierucci et al., 2011; West and Tseng, 2011; Iravani et al., 2012).

Nitric oxide (Calabresi et al., 1999) and dopamine (Calabresi et al., 2000) are essential for the expression of synaptic plasticity, although the effects of nitric oxide may not be restricted to synaptic release sites. nNOS(+) interneurons, via nitric oxide release, have a strong impact on corticostriatal information processing since they exert a modulatory influence on medium spiny neurons (Sardo et al., 2002; West and Grace, 2004) and control the induction of long term depression in medium spiny neurons (Calabresi et al., 1999; Sergeeva et al., 2007). Moreover, nitric oxide acts as a volume transmitter regulating post-synaptic excitability at glutamatergic synapses (Steinert et al., 2008). Nevertheless, the molecular mechanisms of the interactions between the nitric oxide and dopaminergic systems have not been precisely clarified.

Neuromodulators including, nitric oxide play important roles in the physiology of striatal neurons. However, what is the role of the interaction between nitric oxide and dopamine? In the striatum, nitric oxide production has been linked to increased oxidative damage and to both, necrotic and apoptotic neuronal death (Brown and Borutaite, 2002; Kim and Koh, 2002; Ischiropoulos and Beckman, 2003; Przedborski et al., 2003; for a recent review see Brown, 2010). Since dopamine like nitric oxide is redox active (Przedborski et al., 2003), the production of nitric oxide within a TH(+) immunoreactive neuron may provide a unique vulnerability of this cellular subtype compared to other TH(+) immunoreactive neurons that also do not express nNOS(+). On the other hand, cellular nitric oxide demonstrated neuroprotective effects (Wink et al., 1998; Mohanakumar et al., 2002; Sancesario et al., 2004).

The interrelated localization of nNOS(+) and TH(+) containing fibers and cells bodies in the nigrostriatal pathway propose an anatomical link between the two neurotransmitters. Nitric oxide in combination with dopamine may represent suitable targets for therapeutic intervention in neurodegenerative diseases as for example Parkinson’s (for review see Przedborski et al., 2003; Del Bel et al., 2005, 2011; Pierucci et al., 2011; West and Tseng, 2011). The success of this type of strategy is well documented in Padovan-Neto et al. (2009, 2011), Novaretti et al. (2010), Brzozowski et al. (2011), Takuma et al. (2012), and in the recent reviews (Del Bel et al., 2005, 2011; Jenner, 2008; Pierucci et al., 2011; West and Tseng, 2011; Iravani et al., 2012). Future studies will be required to understand how these two factors interact to regulate basal ganglia circuit function.

## Conflict of Interest Statement

The authors declare that the research was conducted in the absence of any commercial or financial relationships that could be construed as a potential conflict of interest.

## Abbreviations

6-OHDA, 6-hydroxy-dopamine; CLSM, confocal laser scanning microscopy; CY3, cyanine-3; DA, dopamine; FITC, fluorescein isothiocyanate; GABA, gamma-aminobutyric acid; l-NAME, N^G^-nitro- l-arginine methyl ester; NADPH, nicotinamide adenine dinucleotide phosphate; NMDA, *N*-Methyl-d-aspartate acid; nNOS, neuronal nitric oxide synthase; NOS, nitric oxide synthase; TH, tyrosine hydroxylase.
